# Th17/Treg imbalance in COPD development: suppressors of cytokine signaling and signal transducers and activators of transcription proteins

**DOI:** 10.1038/s41598-020-72305-y

**Published:** 2020-09-17

**Authors:** Larissa E. F. Silva, Juliana D. Lourenço, Kaique R. Silva, Fernanda Paula R. Santana, Júlia B. Kohler, Alyne R. Moreira, Ana Paula P. Velosa, Carla M. Prado, Rodolfo P. Vieira, Marcelo V. Aun, Iolanda Fátima L. C. Tibério, Juliana T. Ito, Fernanda D. T. Q. S. Lopes

**Affiliations:** 1 Laboratory of Experimental Therapeutics, Department of Clinical Medicine, School of Medicine, University of Sao Paulo, Sao Paulo, SP Brazil; 2 Laboratory of Studies in Pulmonary Inflammation, Department of Bioscience, Federal University of Sao Paulo, Diadema, SP Brazil; 3 Laboratory of Extracelular Matrix, Department of Clinical Medicine, School of Medicine of University of Sao Paulo, Sao Paulo, SP Brazil; 4 Laboratory of Studies in Pulmonary Inflammation, Department of Bioscience, Federal University of Sao Paulo, Santos, SP Brazil; 5 Post-Graduation Program in Bioengineering, Universidade Brasil, Sao Paulo, SP Brazil; 6 Host & Defense Unit, Faculdade Israelita de Ciências da Saúde Albert Einstein, Sao Paulo, SP Brazil; 7 Department of Clinical Medicine, School of Medicine, University of Sao Paulo, Av. Dr. Arnaldo 455 – room 1220, Sao Paulo, SP 01246-903 Brazil

**Keywords:** Immunology, Lymphocytes

## Abstract

Th17/Treg imbalance contributes to chronic obstructive pulmonary disease (COPD) development and progression. However, intracellular signaling by suppressor of cytokine signaling (SOCS) 1 and SOCS3 and the proteins signal transducer and activator of transcription (STAT) 3 and STAT5 that orchestrate these imbalances are currently poorly understood. Thus, these proteins were investigated in C57BL/6 mice after exposure to cigarette smoke (CS) for 3 and 6 months. The expression of interleukin was measured by ELISA and the density of positive cells in peribronchovascular areas was quantified by immunohistochemistry. We showed that exposure to CS in the 3rd month first induced decreases in the numbers of STAT5+ and pSTAT5+ cells and the expression levels of TGF-β and IL-10. The increases in the numbers of STAT3+ and pSTAT3+ cells and IL-17 expression occurred later (6th month). These findings corroborate the increases in the number of SOCS1+ cells in both the 3rd and 6th months, with concomitant decreases in SOCS3+ cells at the same time points. Our results demonstrated that beginning with the initiation of COPD development, there was a downregulation of the anti-inflammatory response mediated by SOCS and STAT proteins. These results highlight the importance of intracellular signaling in Th17/Treg imbalance and the identification of possible targets for future therapeutic approaches.

## Introduction

Chronic obstructive pulmonary disease (COPD) is a chronic inflammatory disease of the airways (obstructive bronchiolitis) with parenchymal destruction (emphysema), that is mainly caused by cigarette smoke (CS) exposure and characterized by persistent airflow obstruction^1^. COPD remains a major cause of morbidity and mortality worldwide and is estimated to become the third most common cause of death by 2020^2^.

T cell-mediated adaptive immunity is deeply involved in the regulation of airway inflammation during COPD development^3,4^, and Th17 cells, a subset of activated CD4 + T cells, are associated with the progression and exacerbation of alveolar destruction via interleukin (IL)-17 release^3–7^. In contrast, regulatory T (Treg) cells are a T cell subtype that is responsible for maintaining immune homeostasis by inhibiting abnormal immune responses and suppressing inflammation^3^.

Clinical and experimental studies have described Th17/Treg imbalances in COPD progression. Sales et al. reported that Treg and IL-10+ cell numbers were decreased in the airways of obstructed smokers compared with healthy smokers and control subjects, whereas all smokers, obstructed or not, showed increases in IL-17+ cell numbers^8^. Cervilha et al. showed in an animal model of CS exposure associated with lipopolysaccharide challenge that the Th17/Treg imbalance led to exacerbation of the inflammatory process^9^.

The differentiation of T cell subsets depends on the microenvironmental stimuli produced by the release of cytokines, which in turn is mediated by intracellular proteins, including signal transducer and activator of transcription (STAT) and suppressor of cytokine signaling (SOCS) proteins^10^.

Th17 cell differentiation occurs in the presence of STAT3, which is activated by the Th17-induced cytokines IL-6, IL-23, and IL-21 and can induce RORγt gene expression. In contrast, STAT5 can directly upregulate Foxp3 gene expression, which induces the development and maintenance of Treg cells^11–13^. Conversely, SOCS1 is defined as an important protein that negatively regulates the interferon-γ–STAT1 pathway^14^, which is essential for Th1 cell differentiation, and SOCS3 is a specific inhibitor of STAT3^15^.

Studies using diaphragm samples^16^ and bronchial biopsies^17^ from COPD patients have demonstrated the important role of the intracellular suppressor protein SOCS in COPD pathogenesis and its potential as a therapeutic target. Chen et al. (2014) identified differentially expressed genes (DEGs) in diaphragm muscle samples from COPD patients and demonstrated that the DEGs IL-6 and SOCS3 directly participated in the JAK/STAT signaling pathway^16^. Springer et al. evaluated SOCS3 gene expression in bronchial mucosa from COPD patients and found that SOCS3 expression was downregulated compared to that in nonsmoker control subjects^17^.

Although the Th17/Treg imbalance has been well described in COPD pathogenesis^6,18–20^, the role of STAT and SOCS expression in T cell differentiation at different phases of COPD development is poorly understood. Thus, we aimed to evaluate the intracellular signaling proteins during COPD development in a long-term CS exposure mouse model.

## Results

### Lung morphometry

**Figure 1:**
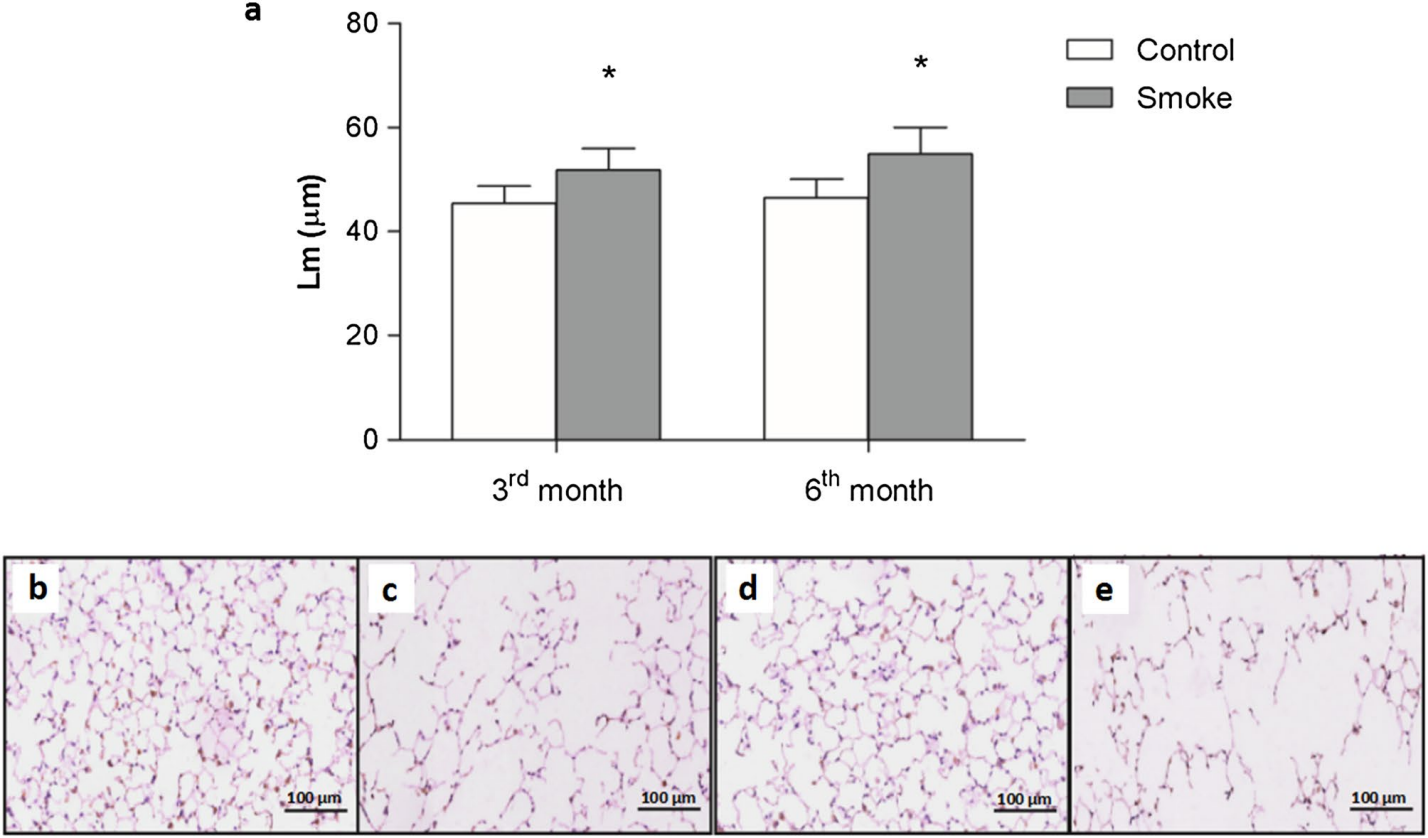
Alveolar enlargement. Lm values of the lung distal parenchyma were determined in the control groups after 3 (n = 9) and 6 (n = 9) months and the smoke groups after 3 (n = 9) and 6 (n = 9) months. The data are shown as the mean ± SE. The smoke groups after 3 and 6 months: (**a**) *p = 0.002 (t-test) and *p = 0.001 (t-test), respectively, compared with their respective controls. (**b**–**e**) Representative photomicrographs of the Lm in the distal parenchyma of the (**b**) control and (**c**) smoke groups in the 3rd month and of the (**d**) control and (**e**) smoke groups in the 6th month are shown at 200 × magnification.

Alveolar enlargement was observed in the parenchyma after the 3rd month of CS exposure. However, there was no difference when the different exposure times were compared (Fig. 1a), as shown in the respective representative photomicrographs of Lm in the distal parenchyma (Fig. 1b–e).

### Th17/Treg imbalance and intracellular signaling

**Figure 2:**
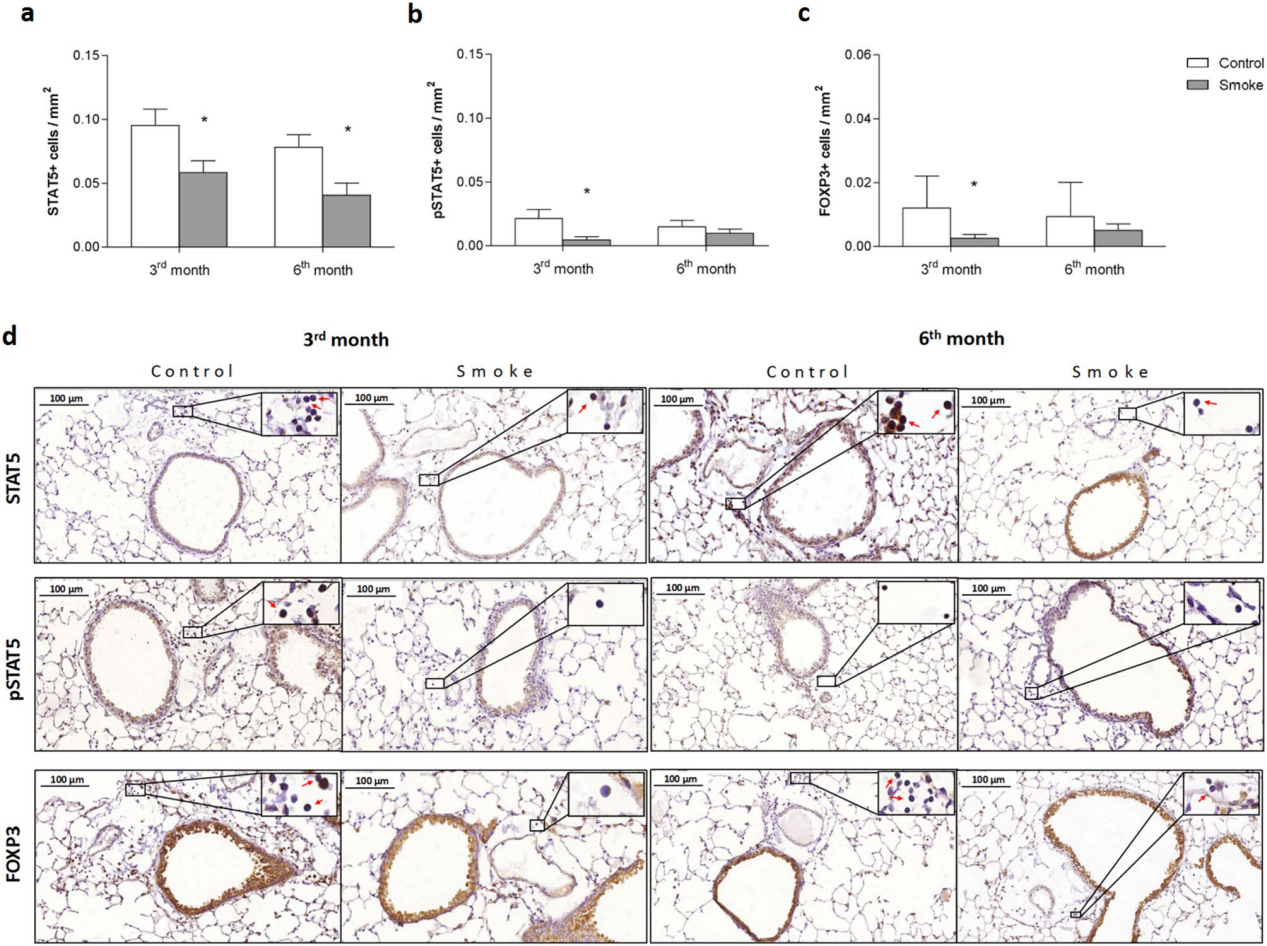
Intracellular signaling and the Treg response. The density of positive cells for (**a**) STAT5, (**b**) pSTAT5 and (**c**) FOXP3 in peribronchovascular areas in the control groups after 3 (n = 6, 5 and 7, respectively) and 6 months (n = 5, 6 and 8, respectively) and in the smoke groups after 3 (n = 7, 5 and 8, respectively) and 6 months (n = 6, 5, and 8, respectively). The data are shown as the mean ± SE. The smoke groups after 3 and 6 months: (**a**) *p = 0.036 (t-test) and *p = 0.022 (t-test), respectively. The smoke groups after 3 months (**b**) *p = 0.032 (Mann–Whitney test) and (**c**) *p = 0.039 (t-test) compared with their controls. (**d**) Representative photomicrographs of peribronchovascular areas are shown at 200 × magnification, and images at 1,000 × magnification are shown in each insert.

There were decreases in the numbers of STAT5+, pSTAT5+, and FOXP3 + cells after 3 months, but only the decrease in the number of STAT5+ cells persisted until 6 months. No significant differences were found between the smoke groups with respect to Treg markers (Fig. 2a–c), as shown in the representative photomicrographs (Fig. 2d).

**Figure 3:**
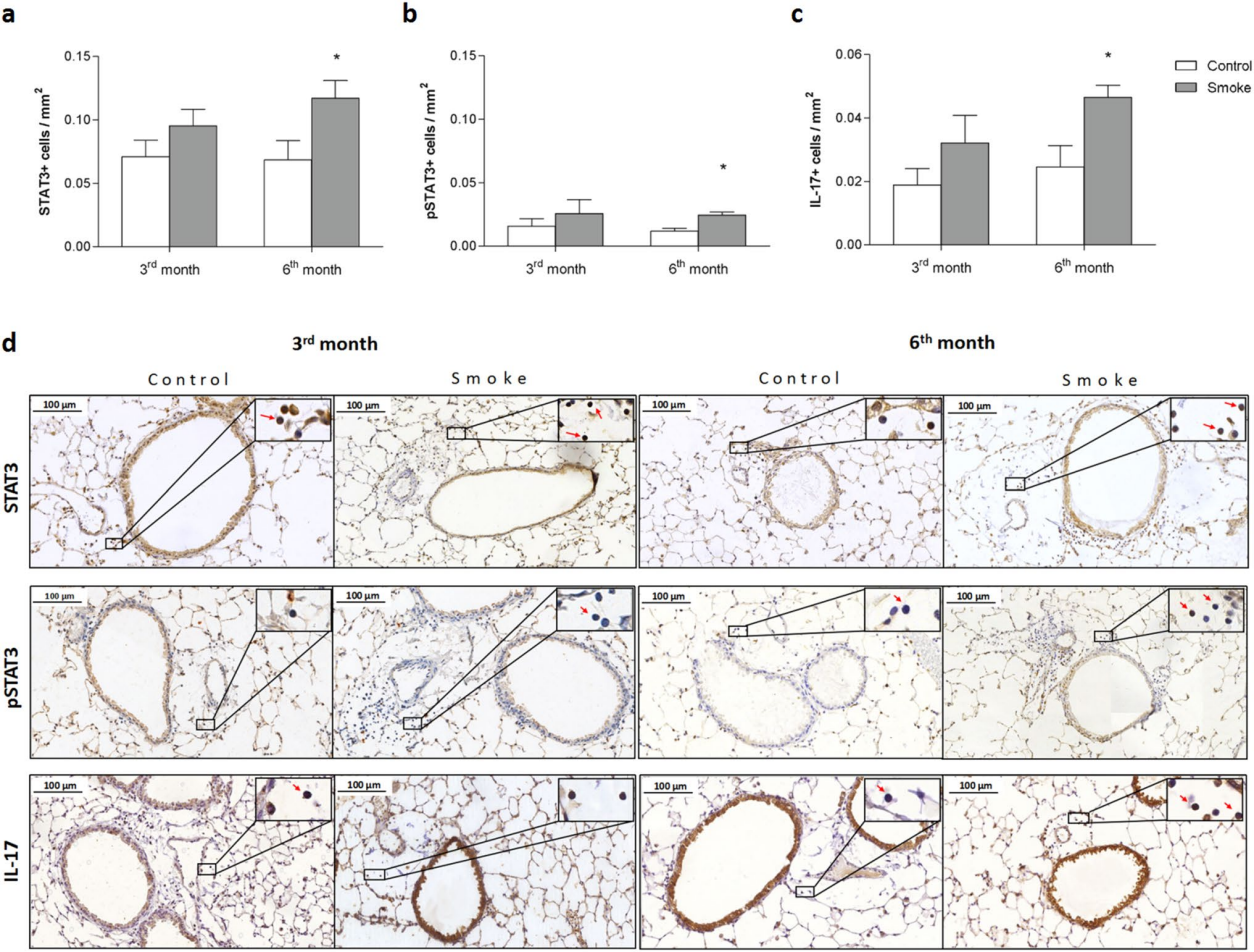
Intracellular signaling and the Th17 response. The density of positive cells for (**a**) STAT3, (**b**) pSTAT3 and (**c**) IL-17 in peribronchovascular areas in the control groups after 3 (n = 9, 9 and 7, respectively) and 6 months (n = 7, 7 and 8, respectively) and in the smoke groups after 3 (n = 8, 8 and 9, respectively) and 6 months (n = 8, 8 and 7, respectively). The data are shown as the mean ± SE. The smoke groups after 6 months: (**a**) *p = 0.028 (t-test), (**b**) *p = 0.031 (t-test) and (**c**) *p = 0.032 (t-test) compared with their controls. (**d**) Representative photomicrographs of peribronchovascular areas are shown at 200 × magnification, and images at 1,000 × magnification are shown in each insert.

In contrast, the smoke group showed an increase in the number of IL-17+ cells in peribronchovascular areas after 6 months of CS exposure, as well as increases in STAT3+ and pSTAT3+ cells at the same time points. No significant differences were observed between the smoke groups with regard to Th17 markers (Fig. 3a–d).

**Figure 4:**
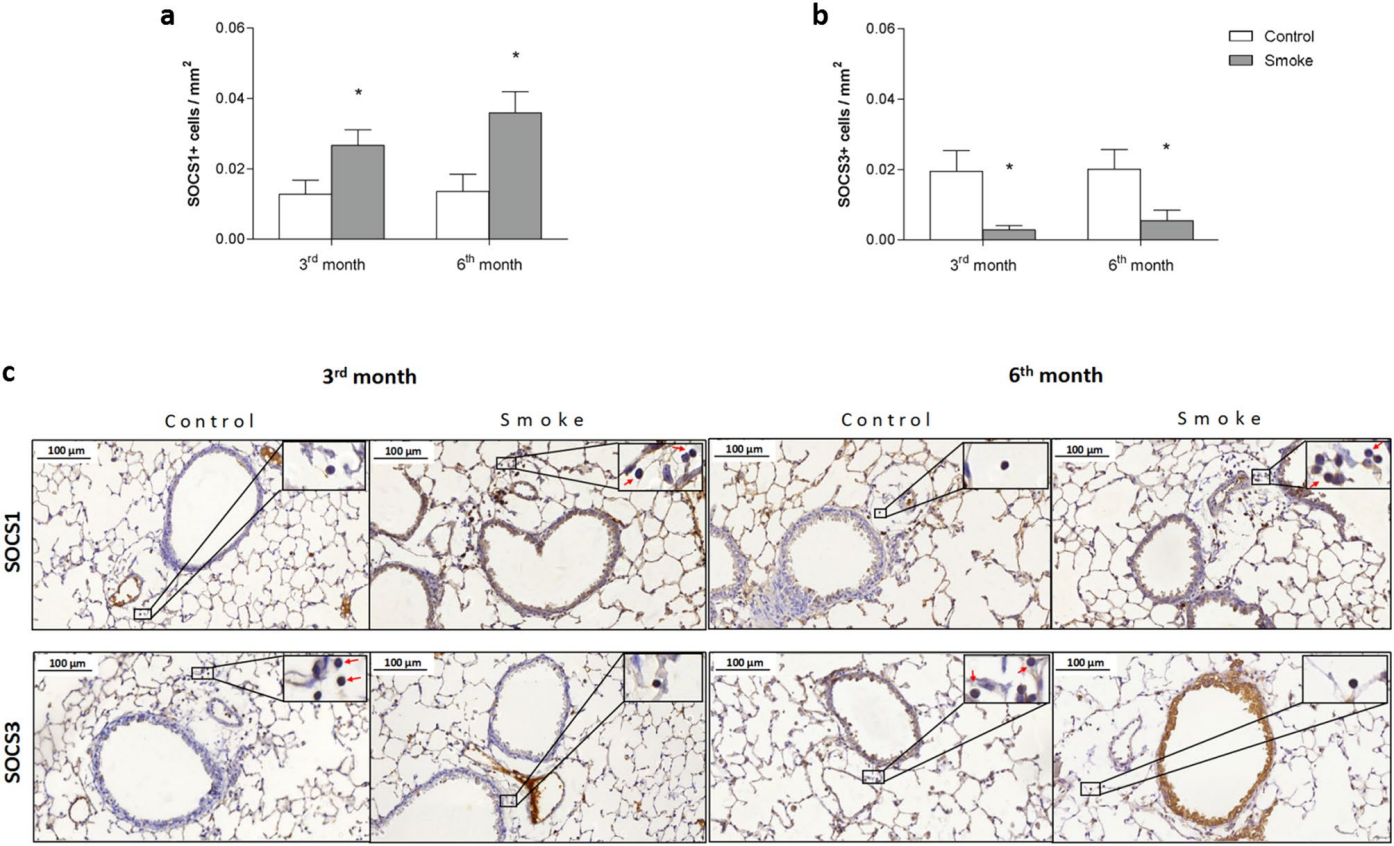
Intracellular SOCS1 and SOCS3 signaling. The density of positive cells for (**a**) SOCS1 and (**b**) SOCS3 in peribronchovascular areas in the control groups after 3 (n = 8 and 8, respectively) and 6 months (n = 8 and 8, respectively) and in the smoke groups after 3 (n = 9 and 8, respectively) and 6 months (n = 6 and 7, respectively). The data are shown as the mean ± SE. The smoke groups after 3 and 6 months: (**a**) *p = 0.035 (t-test) and *p = 0.013 (t-test); (**b**) *p = 0.015 (t-test) and *p = 0.046 (t-test), respectively, compared with their controls. (**c**) Representative photomicrographs of peribronchovascular areas are shown at 200 × magnification, and images at 1,000 × magnification are shown in each insert.

We analyzed suppressor proteins and observed an increase in the number of SOCS1+ cells in peribronchovascular areas at both the 3rd and 6th months of CS exposure, whereas the number of SOCS3+ cells was decreased at the same time points. No significant differences in SOCS1+ and SOCS3+ cell numbers were observed between the smoke groups (Fig. 4a,b), as shown in the representative photomicrographs (Fig. 4c).

### Colocalization of intracellular signaling associated with the Treg and Th17 response

**Figure 5:**
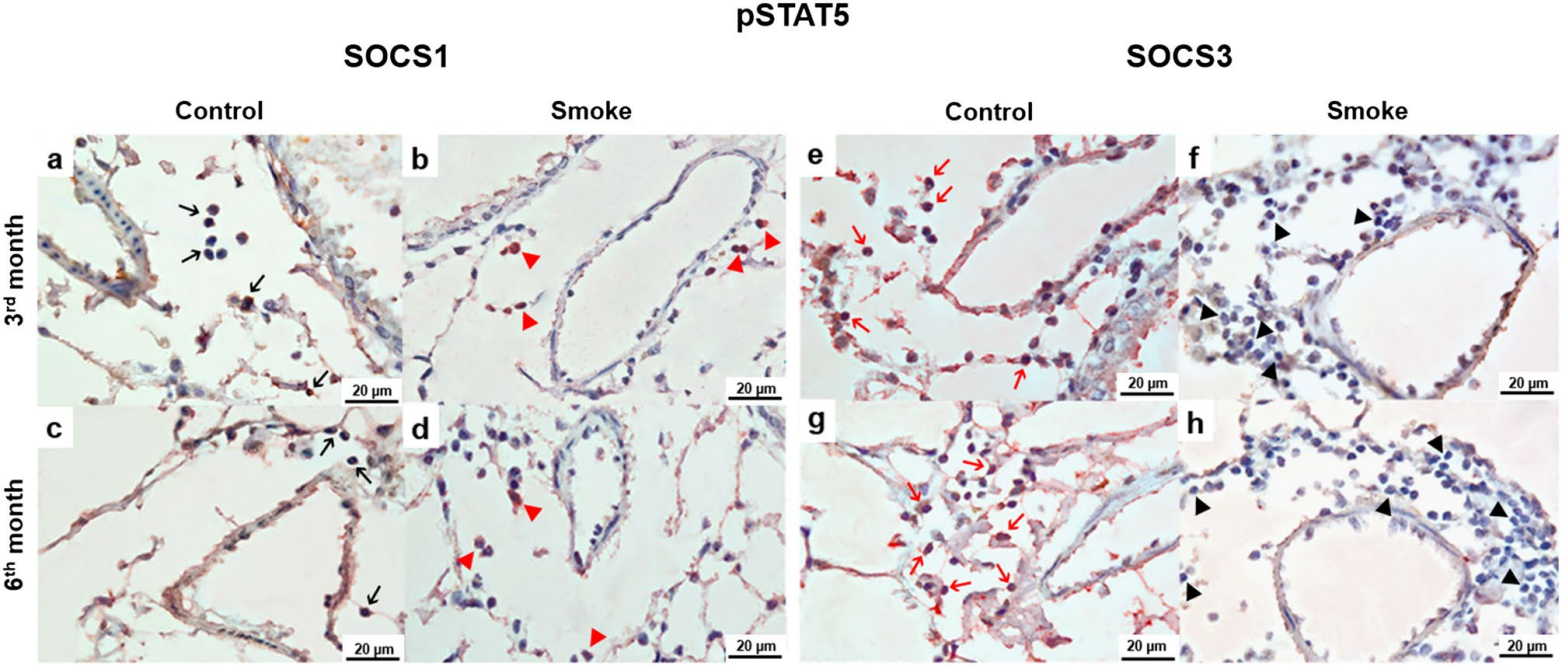
Colocalization of intracellular signaling for the Treg response. Colocalization of infiltrated pSTAT5+ lymphocytes (brown) with (**a**–**d**) SOCS1 (red) and (**e**–**h**) SOCS3 (red) in peribronchovascular areas in the control and smoke groups after 3 and 6 months. (**a**, **c**) In the control groups, there was a prevalence of pSTAT5+ lymphocytes (black arrows), (**e**, **g**) whereas the red arrows indicate colocalization with SOCS3. (**b**, **d**) The smoke groups exhibited SOCS1+ lymphocytes (red arrowheads) and (**f**, **h**) weak signals for both pSTAT5 and SOCS3 (black arrowheads). (**a**–**h**) Representative photomicrographs of peribronchovascular areas are shown at 1,000 × magnification.

**Figure 6:**
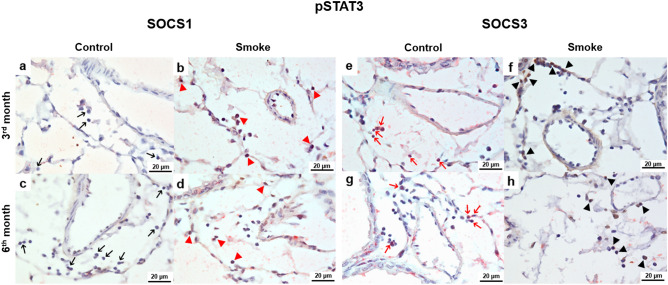
Colocalization of intracellular signaling for the Th17 response. Colocalization of infiltrated pSTAT3+ lymphocytes (brown) with (**a**–**d**) SOCS1 (red) and (**e**–**h**) SOCS3 (red) in peribronchovascular areas in the control and smoke groups after 3 and 6 months. (**a**, **c**) The control groups showed weak signals for pSTAT3 and SOCS1 (black arrows) and (e and g) increased numbers of SOCS3+ lymphocytes (red arrows). (**b**, **d**) The smoke groups demonstrated double staining for pSTAT3 and SOCS1 (red arrowheads) and (**f**, **h**) increased numbers of pSTAT3+ lymphocytes (black arrowheads). (**a**–**h**) Representative photomicrographs of peribronchovascular areas are shown at 1,000 × magnification.

The double-staining analysis showed the immunohistochemical findings. The double-staining images showed the colocalization of lymphocytes that were positive for pSTAT5 and SOCS1 (Fig. 5a–d) or SOCS3 (Fig. 5e–h). There was an increase in pSTAT5 expression in the control groups after 3 and 6 months (Fig. 5a,c) and colocalization of pSTAT5 with SOCS3 compared with those of the smoke groups (Fig. 5e,g). In contrast, the smoke groups exhibited SOCS1+ lymphocytes (Fig. 5b,d) and weak signals for both pSTAT5 and SOCS3 (Fig. 5f,h). We also performed double staining of lymphocytes for pSTAT3 and SOCS1 (Fig. 6a–d) or SOCS3 (Fig. 6e–h). The control groups showed weak signals for pSTAT3 and SOCS1 (Fig. 6a,c) and increased numbers of SOCS3+ lymphocytes (Fig. 6e,g). In the smoke groups, colocalization of pSTAT3 with SOCS1 was most evident (Fig. 6b,d), and there was an increase in pSTAT3 compared with that of the control groups (Fig. 6f,h).

### Cytokine levels in lung homogenates

**Figure 7:**
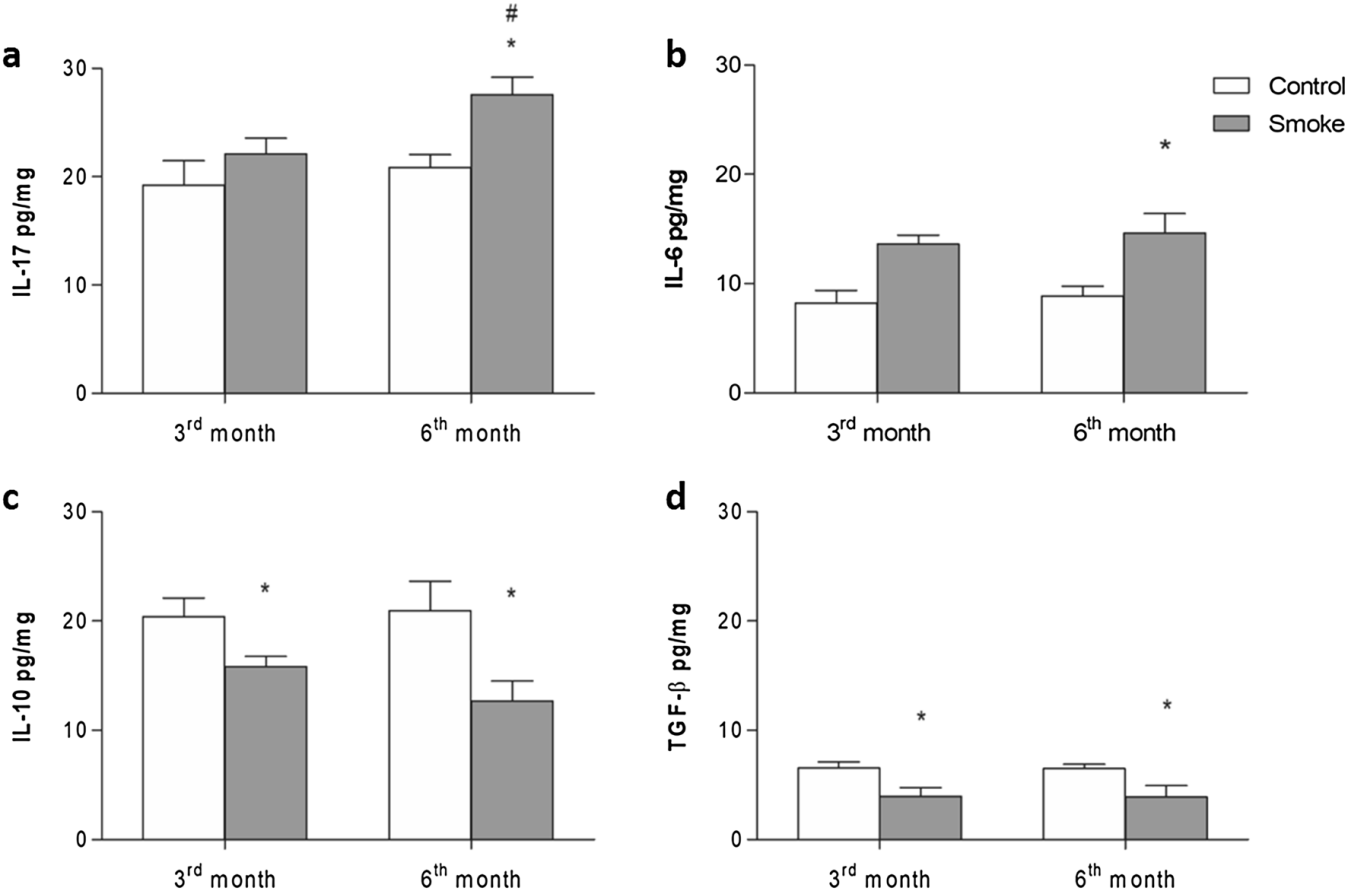
Cytokine levels. The levels of (**a**) IL-17, (**b**) IL-6, (**c**) IL-10 and (**d**) TGF-β in lung homogenates were measured in the control groups after 3 (n = 7, 8, 7, and 5, respectively) and 6 (n = 6, 8, 7, and 5, respectively) months and in the smoke groups after 3 (n = 6, 5, 5, and 5, respectively) and 6 (n = 5, 7, 8, and 5, respectively) months and are presented as the mean ± SE. Smoke group after 6 months: (**a**) *p = 0.007 (t-test) compared with their control and #p = 0.032 (t-test) compared with the smoke group at 3 months and (**b**) *p = 0.004 (Mann–Whitney test). In the smoke groups after 3 and 6 months: (**c**) *p = 0.030 (Mann–Whitney test) and *p = 0.040 (Mann–Whitney test) and (**d**) *p = 0.029 (Mann–Whitney test) and *p = 0.016 (Mann–Whitney test), respectively, compared with their controls.

There was an increase in the level of IL-17 in the lungs of the smoke group compared with the control group only at 6 months of CS exposure, and this increase was higher in the group that was exposed to CS for 6 months than in the group that was exposed for 3 months. Additionally, we demonstrated an increased level of IL-6 in the 6th month of CS exposure (Fig. 7a,b). In contrast, we observed decreases in IL-10 and TGF-β levels in lung homogenates after 3 and 6 months of CS exposure. However, no significant differences were observed between the smoke groups with different exposure times (Fig. 7c,d).

### Correlations between Th17 and Treg responses and intracellular signaling

**Figure 8:**
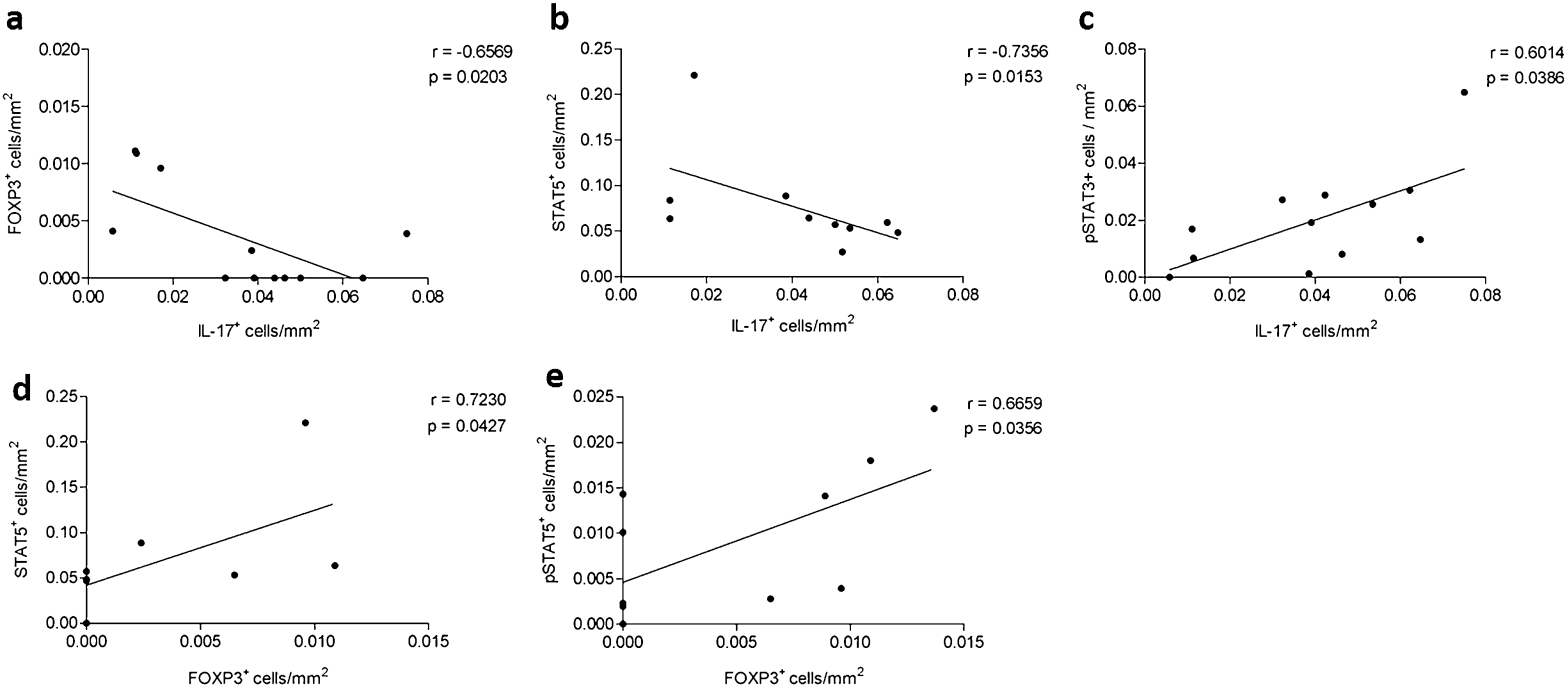
Correlation analysis. Correlations between the density of (**a**) IL-17+ cells and FOXP3+ cells, (**b**) IL-17+ cells and STAT5+ cells, (**c**) IL-17+ cells and pSTAT3+ cells, (**d**) FOXP3+ cells and STAT5+ cells and (**e**) FOXP3+ cells and pSTAT5+ cells among all smoke groups. The data were analyzed by the Spearman’s rank correlation coefficient.

We analyzed all peribronchovascular areas of CS-exposed mice and found a negative correlation between the number of IL17+ cells and FOXP3+ cells, supporting the previously identified Th17/Treg imbalance (Fig. 8a). Furthermore, the number of IL-17+ cells was inversely correlated with the number of STAT5+ cells and was also correlated with the number of pSTAT3+ cells (Fig. 8b,c). In addition, we observed a positive correlation between the number of FOXP3+ cells and STAT5+ and pSTAT5+ cells (Fig. 8d,e).

## Discussion

Our results showed that decreases in intracellular signaling for Treg cell differentiation and IL-10 release in peribronchovascular areas of the lung occur prior to the modulation Th17 signaling in a CS-induced model of COPD. We also found a negative correlation between the Th17 response and FOXP3+  and STAT5+ cell numbers. Decreases in the numbers of STAT5+ and pSTAT5+ cells and IL-10 expression occurred beginning at the early stages of COPD development; only after these events occurred there were increases in STAT3+ and pSTAT3+ cell numbers and IL-17 expression.

FOXP3 is a transcription factor expressed in Treg cells that is essential for their development and the maintenance of their suppressive activity^21,22^, and TGF-β is the cytokine responsible for the activation of this transcription factor^23^.

Previously, in a CS-induced model of COPD, we showed reductions in IL-10+, TGF-β+ and Treg cell numbers in peribronchovascular areas associated with worsening lung functions beginning with the initial events of COPD development^24^. Although we observed that the numbers of Treg cells in mice exposed to CS returned to numbers similar to those in the control group in later stages, the reductions in IL-10+ and TGF-β+ cell numbers remained until the end of the protocol, suggesting a worsening of the suppression activity of Treg cells. In the present study, we observed decreases in FOXP3+ and pSTAT5+ cell numbers beginning at the 3rd month, and although we did not find significant differences at the 6th month, fewer positive cells were observed in the CS group than in the control group. Moreover, we demonstrated positive correlations between the numbers of FOXP3+, STAT5+ and pSTAT5+ cells.

The importance of STAT5 signaling in FOXP3 expression and Treg cell activity was addressed by Passerini et al., who showed that STAT5-activating cytokines induce FOXP3 expression in vitro in activated human effector T cells, suggesting a critical role for STAT5 in maintaining the suppressive function of Treg cells^25^. TGF-β1 is also essential for FOXP3 expression and the regulatory function of peripheral Treg cells^26^; however, TGF-β1 can inhibit T cell differentiation in the presence of inflammatory cytokines such as IL-6 and IL-21^27^.

The decrease in IL-10, the effector molecule of Treg cells, from the beginning of COPD development in this model could be due to the decreased numbers of both pSTAT5+ and FOXP3+ cells. However, further studies will be necessary to better understand how STAT5 phosphorylation could interfere with IL-10 production through insufficient FOXP3 expression in Treg cells.

Although the decrease in the anti-inflammatory response and the enlargement of airway spaces were well defined beginning at the 3rd month, Th17 skewing was detected only at the 6th month, when we detected increased numbers of STAT3+ and pSTAT3+ cells, as well as increased IL-6 levels. Ruwanpura et al. demonstrated in mice that IL-6 induced the activation of STAT3, leading to Th17 cell differentiation during the inflammatory process^28^. Additionally, elevated levels of IL-6 in both sputum^29^ and lung samples from COPD patients^30^ and the importance of the STAT3 pathway have been shown in the advanced stages of this disease. Moreover, Duan et al. showed an increase in IL-17-producing T cells and IL-6 levels accompanied by the upregulation of the mRNA expression of RORγt, an important transcription factor of Th17 cells in long-term CS-exposed mice^20^.

The Th17 response has been extensively described as playing a pivotal role in autoimmune diseases^31^ and more recently in COPD development and progression^32^. Ponce-Garrez et al. showed an association between a polymorphism in the cytokine IL-17A and lung disease development. Additionally, this group found increased IL-17 serum levels in all smokers, independent of COPD development^32^. These results support our previous findings that showed significantly increased IL-17+ cell density in the small airways of smokers with or without COPD. However, in a study performed by Sales et al., only obstructed smokers (COPD) showed reductions in Treg cells and IL-10+ cells in small airways^8^. Our present data are in agreement with these previous studies, suggesting that the progression of the Th17 response was mitigated by a failure in IL-10 release, which worsens inflammatory control.

Regarding the analysis of suppressor of cytokine signaling proteins, we observed a decrease in SOCS3 levels from the beginning of the protocol, along with an increase in IL-6 expression. Crocker et al. (2003) showed that a lack of SOCS3 induced increased IL-6 signaling in hepatocytes and macrophages in culture^33^. Consistent with this finding, SOCS3 expression in diaphragm samples^16^ and bronchial biopsies^17^ from COPD patients was decreased compared to that in controls.

In contrast, we showed an increase in SOCS1 expression at the 3rd month, which persisted until the end of the protocol. Previous studies have shown that SOCS1 promotes Th17 differentiation by regulating SOCS3 levels and by blocking INF-γ signaling, thus preventing Th1 polarization. Although it is poorly understood, SOCS1 may also negatively regulate the production of FOXP3+ cells in the thymus^34,35^, which is partially consistent with our findings.

To our knowledge, this is the first study to analyze the involvement of STAT and SOCS proteins in Th17/Treg differentiation during COPD development. These findings reinforce previous experimental and clinical results that showed the injurious effects of CS exposure in lung inflammation control leading to COPD development.

Some limitations of this study need to be acknowledged. First, as we used an animal model, we did not completely translate these results for humans. Additionally, further investigation will be necessary to elucidate how different Treg phenotypes interfere in IL-10 release, as well as in the anti-inflammatory immune response during CS exposure.

## Conclusion

We showed in a CS-induced model that a STAT and SOCS protein-mediated failure in inflammatory control mitigates Th17/Treg imbalance, leading to COPD development. These results are important for identifying possible targets for future investigations in therapeutic approaches.

## Materials and methods

### Experimental groups

Eighty male C57BL/6 mice (aged 6–8 weeks and weighing 20–25 g) were used in two protocols (enzyme-linked immunosorbent assay (ELISA) and immunohistochemistry), with forty mice divided into groups exposed to CS for 3 and 6 months. The control groups were maintained under filtered air conditions for the same periods of time (n = 10 for each time point). All animals received humane care in compliance with the Guide for the Care and Use of Laboratory Animals published by the US National Institutes of Health (NIH Publication No. 85–23, revised 1996). Our protocol was approved by the Ethics Committee of the School of Medicine of the University of Sao Paulo (protocol number 070/16; Sao Paulo, Brazil).

### CS exposure protocol

To induce COPD, the animals were exposed to CS as previously described^24,36^. The mice were maintained in the CS environment for 30 min, twice per day, 5 days per week for a period of 3 and 6 months. The flow rate was set such that the carbon monoxide (CO) levels ranged from 250 to 350 parts per million. We used 12 ± 1 commercially filtered cigarettes (0.8 mg of nicotine, 10 mg of tar, and 10 mg of CO per cigarette) per exposure, resulting in a total particulate matter concentration of 354.8 ± 50.3 μg/m^3^/day.

### Lung preparation

At the end of the protocol, the mice were intraperitoneally anesthetized with thiopental (70 mg/kg) and euthanized by transecting the abdominal aorta as previously described^24^. The lungs were removed and fixed with 10% formaldehyde infused through the trachea at a constant pressure of 20 cm H_2_O for 24 h. The lungs were embedded in paraffin and cut into 5 μm coronal sections.

### Morphometry

To evaluate the mean linear intercept (Lm), an indicator of the mean alveolar diameter^37^, lung tissue sections were stained with hematoxylin and eosin (H&E). We performed the Lm measurements by attaching an eyepiece with 50 lines and 100 points with a known area (62.500 μm^2^ at 400 × magnification) to the ocular lens of the microscope^38^. For each animal, 20 nonoverlapping fields in the distal lung parenchyma were assessed at 400 × magnification. Lm quantification was determined by counting the number of times that the reticle lines intercepted the alveolar walls. After obtaining the mean values of the fields for each histological slide, the Lm was calculated by the following equation:

$${\text{Lm }} = {\text{ Ltotal}}/{\text{NI}},$$

where Ltotal is the sum of all reticle segments and NI is the average number of times that the lines intersected the alveolar walls. The Lm values are presented in micrometers (μm).

### Immunohistochemistry

Lung tissue sections were immunostained with the following primary antibodies, which are listed in Table 1: FOXP3, IL-17, STAT5, p-STAT5, STAT3, p-STAT3, SOCS3 and SOCS1. Species-specific secondary antibodies in conjunction with a Vector ABC kit (Vector Laboratories, CA, USA) were used for the reactions. All sections were stained using the chromogen 3,3′-diaminobenzidine (DAB, Sigma-Aldrich, MO, USA) and counterstained with Harris’s hematoxylin (Merck, Darmstadt, Germany).

**Table 1:** Description of antibodies used for immunohistochemistry.

Antibody	Isotype	Dilution	Code	Company
FOXP3	Rabbit IgG	1:700	ab54501	Abcam, Cambridge, UK
IL-17	Rabbit IgG	1:200	ab79056	Abcam, Cambridge, UK
STAT5	Rabbit IgG	1:750	sc-836	Santa Cruz, CA, USA
p-STAT5	Goat IgG	1:100	sc-12893	Santa Cruz, CA, USA
STAT3	Rabbit IgG	1:5,000	D1B2J	Cell Signaling Technology MA, USA
p-STAT3	Rabbit IgG	1:150	D3A7	Cell Signaling Technology MA, USA
SOCS3	Mouse IgG	1:200	ab78341	Abcam, Cambridge, UK
SOCS1	Mouse IgG	1:200	ab211288	Abcam, Cambridge, UK

The stained lung tissue sections were scanned using a high-resolution digital scanner (Pannoramic Scan, 3D Histech, Budapest, Hungary), and the densities of specific cells in the peribronchovascular area were calculated using Panoramic Viewer software 1.5 (3D Histech, Budapest, Hungary). For this analysis, 5 to 10 peribronchovascular areas at 200 × magnification were selected, and the positive lymphocytes were counted and subsequently divided by the total corrected area (represented by the airway and peribronchovascular space, after subtraction of the blood vessel and airway lumen areas). The results are expressed as cells/mm^2^.

Double immunohistochemical staining was performed to analyze the colocalization of pSTAT5 with SOCS1 or SOCS3 in the peribronchovascular areas. First, the lung tissue sections were incubated with anti-pSTAT5 and stained using the chromogen DAB (Sigma, MO, USA), as previously described. Then, the sections were incubated with anti-SOCS1 or anti-SOCS3 following the equivalent protocol and stained using an immunoalkaline phosphatase procedure and a PermaRed reagent (Diagnostic BioSystems, Pleasanton, CA). The same procedure was performed to analyze cells for pSTAT3 colocalization with SOCS1 or SOCS3.

The lung images were captured using an Olympus BX51 microscope (Tokyo, Japan) and ImageJ software (US National Institutes of Health) at 1,000 × magnification.

### Quantification of the total protein and cytokine levels in pulmonary tissue

Using a polytron PTA 20S (Brinkmann Instruments, model PT 10/35, Westbury, NY, USA), the right lungs were homogenized in tubes containing 1 mL of protein lysis extraction buffer and a cocktail of protease and phosphatase inhibitors (2 mM Tris, 150 mM NaCl, Nonidet P-40 2%, glycerol 10%, 20 mM sodium fluoride, 0.2% SDS, 0.5% sodium deoxycholate, 1 mM sodium orthovanadate, 1 mM aprotinin and 1 mM PMSF). Subsequently, each sample was mixed with 100 μL of Triton-X (Sigma-Aldrich, Merck KgaA, Darmstadt, Germany), incubated on ice for 15 min and centrifuged at 12,000×*g* for 15 min at 4 °C to collect the supernatant. The total protein concentration was measured by the Bradford method (Protein Assay, Bio-Rad, California, USA) using a known protein curve of bovine serum albumin (BSA, Sigma-Aldrich, MO, USA) at concentrations of 0.2, 0.4, 0.6, 0.8 and 1.0 mg/mL and distilled water (blank) in duplicate, and the protein extract was used at a 1:40 dilution in distilled water. The absorption spectra at approximately 450 nm were recorded for all samples using a spectrophotometer (Epoch-Biotek, Vermont, USA) and GEN 5.1.1.1 software (Biotek, VT, USA). All spectroscopic data were analyzed using GraphPad InStat software (version 3.0, CA, USA) and are expressed as mg/mL.

The levels of IL-17, IL-6, IL-10 and transforming growth factor-beta (TGF-β) were quantified by ELISA (enzyme-linked immunosorbent assay) using Duo Set kits (R&D Systems, Minneapolis, MN, USA) specific for each cytokine, as shown in Table 2. The reaction was measured at 450 nm in an M2 spectrophotometer (SpectraMax L, Molecular Devices). The sample concentrations were calculated from the standard curves, which were generated with the recombinant cytokines and GraphPad InStat software (version 3.0, CA, USA). The data were converted and are expressed as pg/mg.

**Table 2:** Description of the antibodies used in ELISA.

Antibody	Code	Company
IL-17	M1700	R&D Systems, Minneapolis, MN, USA
IL-6	M6000B	
IL-10	M1000B	
TGF- β	MGD150	

### Statistical analysis

The statistical analysis was performed using GraphPad Prism 5.0 (GraphPad, San Diego, CA, USA). The normality of the data distribution was verified with the Shapiro–Wilk test, and the data are presented as the mean ± standard error (SE). The data were analyzed using t-tests for parametrically distributed data or Mann–Whitney tests for nonparametrically distributed data.

Because we did not observe significant differences between the control groups at different time points, we also used t-tests or Mann–Whitney tests to compare the different smoke groups. Correlation analyses were performed using the Spearman’s test. The differences were considered significant at P < 0.05.

### Ethics committee approval

The present study was approved by the Ethics Committee on Human and Animal Research of the School of Medicine of the University of Sao Paulo, Sao Paulo, Brazil (Protocol Number 070/16).

## Data Availability

Data supporting the findings of this study are available within the article.

## References

[1] 1Global Initiative for Chronic Obstructive Lung Disease 2020 (GOLD 2020). The Global Strategy for the Diagnosis, Management and Prevention of COPD (updated 2020).

[2] Mathers CD, Loncar D (2015). Projections of global mortality and burden of disease from 2002 to 2030. PLoS Med..

[3] Brusselle GG, Joos GF, Bracke KR (2011). New insights into the immunology of chronic obstructive pulmonary disease. Lancet.

[4] Zhang JC (2016). TGF-beta/BAMBI pathway dysfunction contributes to peripheral Th17/Treg imbalance in chronic obstructive pulmonary disease. Sci. Rep..

[5] Eppert BL, Wortham BW, Flury JL, Borchers MT (2013). Functional characterization of T cell populations in a mouse model of chronic obstructive pulmonary disease. J. Immunol..

[6] Wang H (2015). Imbalance of peripheral blood Th17 and Treg responses in patients with chronic obstructive pulmonary disease. Clin. Respir. J..

[7] Zhao P (2018). Restoring Th17/Treg balance via modulation of STAT3 and STAT5 activation contributes to the amelioration of chronic obstructive pulmonary disease by Bufei Yishen formula. J. Ethnopharmacol..

[8] Sales DS (2017). Regulatory T-Cell distribution within lung compartments in COPD. COPD.

[9] Cervilha DAB (2019). The Th17/Treg cytokine imbalance in chronic obstructive pulmonary disease exacerbation in an animal model of cigarette smoke exposure and lipopolysaccharide challenge association. Sci. Rep..

[10] Yoshimura A, Suzuki M, Sakaguchi R, Hanada T, Yasukawa H (2012). SOCS, inflammation, and autoimmunity. Front. Immunol..

[11] Zorn E (2006). IL-2 regulates FOXP3 expression in human CD4+CD25+ regulatory T cells through a STAT-dependent mechanism and induces the expansion of these cells in vivo. Blood.

[12] Zhou L (2008). TGF-beta-induced Foxp3 inhibits T(H)17 cell differentiation by antagonizing RORgammat function. Nature.

[13] Sheng W (2014). STAT5 programs a distinct subset of GM-CSF-producing T helper cells that is essential for autoimmune neuroinflammation. Cell Res..

[14] Yoshimura A, Naka T, Kubo M (2007). SOCS proteins, cytokine signalling and immune regulation. Nat. Rev. Immunol..

[15] Yamamoto K, Yamaguchi M, Miyasaka N, Miura O (2003). SOCS-3 inhibits IL-12-induced STAT4 activation by binding through its SH2 domain to the STAT4 docking site in the IL-12 receptor beta2 subunit. Biochem. Biophys. Res. Commun..

[16] Chen W, Hong YQ, Meng ZL (2014). Bioinformatics analysis of molecular mechanisms of chronic obstructive pulmonary disease. Eur. Rev. Med. Pharmacol. Sci..

[17] Springer J (2013). Transcriptional down-regulation of suppressor of cytokine signaling (SOCS)-3 in chronic obstructive pulmonary disease. J. Occup. Med. Toxicol..

[18] Jin Y (2014). Treg/IL-17 ratio and Treg differentiation in patients with COPD. PLoS ONE.

[19] Li H (2015). Disruption of th17/treg balance in the sputum of patients with chronic obstructive pulmonary disease. Am. J. Med. Sci..

[20] Duan MC (2016). Infiltration of IL-17-producing T cells and Treg cells in a mouse model of smoke-induced emphysema. Inflammation.

[21] Chen W, Konkel JE (2010). TGF-beta and 'adaptive' Foxp3(+) regulatory T cells. J. Mol. Cell Biol..

[22] Ohkura N, Hamaguchi M, Sakaguchi S (2011). FOXP3+ regulatory T cells: control of FOXP3 expression by pharmacological agents. Trends Pharmacol. Sci..

[23] de Lafaille MAC, Lafaille JJ (2009). Natural and adaptive foxp3+ regulatory T cells: more of the same or a division of labor?. Immunity.

[24] Ito JT (2019). Th17/Treg imbalance in COPD progression: A temporal analysis using a CS-induced model. PLoS ONE.

[25] Passerini L (2008). STAT5-signaling cytokines regulate the expression of FOXP3 in CD4+CD25+ regulatory T cells and CD4+CD25- effector T cells. Int. Immunol..

[26] Marie JC, Letterio JJ, Gavin M, Rudensky AY (2005). TGF-beta1 maintains suppressor function and Foxp3 expression in CD4+CD25+ regulatory T cells. J. Exp. Med..

[27] Korn T, Bettelli E, Oukka M, Kuchroo VK (2009). IL-17 and Th17 cells. Annu. Rev. Immunol..

[28] Ruwanpura SM (2014). IL-6/Stat3-driven pulmonary inflammation, but not emphysema, is dependent on interleukin-17A in mice. Respirology.

[29] Eickmeier O (2010). Sputum biomarker profiles in cystic fibrosis (CF) and chronic obstructive pulmonary disease (COPD) and association between pulmonary function. Cytokine.

[30] Yew-Booth L (2015). JAK-STAT pathway activation in COPD. Eur. Respir. J..

[31] Eskandari-Nasab E, Moghadampour M, Tahmasebi A (2017). Meta-analysis of risk association between interleukin-17A and F gene polymorphisms and inflammatory diseases. J. Interferon Cytokine Res..

[32] Ponce-Gallegos MA (2020). Genetic variants in IL17A and serum levels of IL-17A are associated with COPD related to tobacco smoking and biomass burning. Sci. Rep..

[33] Croker BA (2003). SOCS3 negatively regulates IL-6 signaling in vivo. Nat. Immunol..

[34] Lu LF (2009). Foxp3-dependent microRNA155 confers competitive fitness to regulatory T cells by targeting SOCS1 protein. Immunity.

[35] Zhan Y (2009). SOCS1 negatively regulates the production of Foxp3+ CD4+ T cells in the thymus. Immunol. Cell Biol..

[36] Toledo AC (2012). Aerobic exercise attenuates pulmonary injury induced by exposure to cigarette smoke. Eur. Respir. J..

[37] Margraf LR, Tomashefski JF, Bruce MC, Dahms BB (1991). Morphometric analysis of the lung in bronchopulmonary dysplasia. Am. Rev. Respir. Dis..

[38] Weibel ER (1963). Principles and methods for the morphometric study of the lung and other organs. Lab. Invest..

